# Data on the thermo-fluid simulation of open-cathode fuel cell stack depending on the location of the oxidizer/cooling supply system

**DOI:** 10.1016/j.dib.2020.105771

**Published:** 2020-05-27

**Authors:** Evgeny Anisimov, Nikita Faddeev, Nina Smirnova

**Affiliations:** Platov South-Russian State Polytechnic University (NPI), 132 Prosveschenia Str., Novocherkassk 346428 (Russia)

**Keywords:** Fuel cell, bipolar plate, design, finite element method

## Abstract

The content of this paper provides simulation data of the distribution of temperature fields, and oxidizer/cooling agent (air) flows in dependence with location of the oxidizer/cooling supply system in open-cathode polymer electrolyte membrane fuel cell (PEMFC) stack. The finite element method in Solid Works Simulation and Solid Works Flow Simulation software were used for bipolar plate strength calculation and thermo-fluid simulation of PEMFC stack with forced-air convection. The simulations were carried out for two variants of the oxidizer/cooling supply system location - at the entrance to the fuel cell stack (air injection) and at the outlet of the fuel cell stack (air intake).

Specifications table

**Table:** 

Subject	Design
Specific subject area	Open-cathode polymer electrolyte fuel cell system
Type of data	Figure, Table
How data were acquired	Finite element analysis. Instruments: Solid Works Flow Simulation and Solid Works Simulation software program.
Data format	Fld, geom, info Analyzed
Parameters for data collection	Open-cathode fuel cells stack; Number of fuel cells in stack - 30 units; Fuel cell consists of a membrane electrode assembly and bipolar plate; Titanium bipolar plate (size 40 • 237 mm) consist of two parts; Cathode part of the bipolar plate has a channels configuration - width 1.75 mm; height 1 mm; pitch - 1.25 mm; Anode part of the bipolar plate has smooth configuration; Oxidizing / cooling agent – air; Oxidizing / cooling agent flow rate - 0.007 m^3^/s; Heat emission as a result of fuel oxidation - 50% of the nominal fuel cell stack power; Heat transfer coefficient from FC stack to outside – 0; Atmospheric pressure - 101.325 Pa; Ambient temperature - 20°C.
Description of data collection	Solid Works Flow Simulation and Solid Works Simulation software program complex were used to perform calculations. The calculations were performed as follows: a three-dimensional model was modelled with the proposed configuration of bipolar plate geometric parameters, after which a strength calculation of bipolar plate was performed. Then, the thermo-fluid simulation of PEMFC stack with forced-air convection was calculated under the conditions of the oxidizing / cooling agent supply.
Data source location	Institution: Platov South-Russian State Polytechnic University (NPI) City: Novocherkassk Country: Russia
Data accessibility	Repository name: Mendeley Data Data identification number: doi:10.17632/32n8cc4syr.2 Direct URL to data: https://data.mendeley.com/datasets/32n8cc4syr/2

## Value of the data

• The data are important in the research area of design of fuel cells. This data can be used to develop fundamentally new designs of metal bipolar plates for open-cathode proton-exchange membrane fuel cell.
• The data can be most useful for the researchers, companies and corporations involved in the development of energy systems based on fuel cells and for energy supply of unmanned aerial vehicles specifically.
• Data of the distribution of temperature fields and gas flows of oxidizing / cooling agent will help to manager the processes in the fuel cell stack. The data obtained will help to optimize the mass-dimensional characteristics of open-cathode PEMFC and increase their specific characteristics.
• The data can be used in the design of the energy systems for energy supply of unmanned aerial vehicles.

## 1 Data Description

**Fig. 1:**
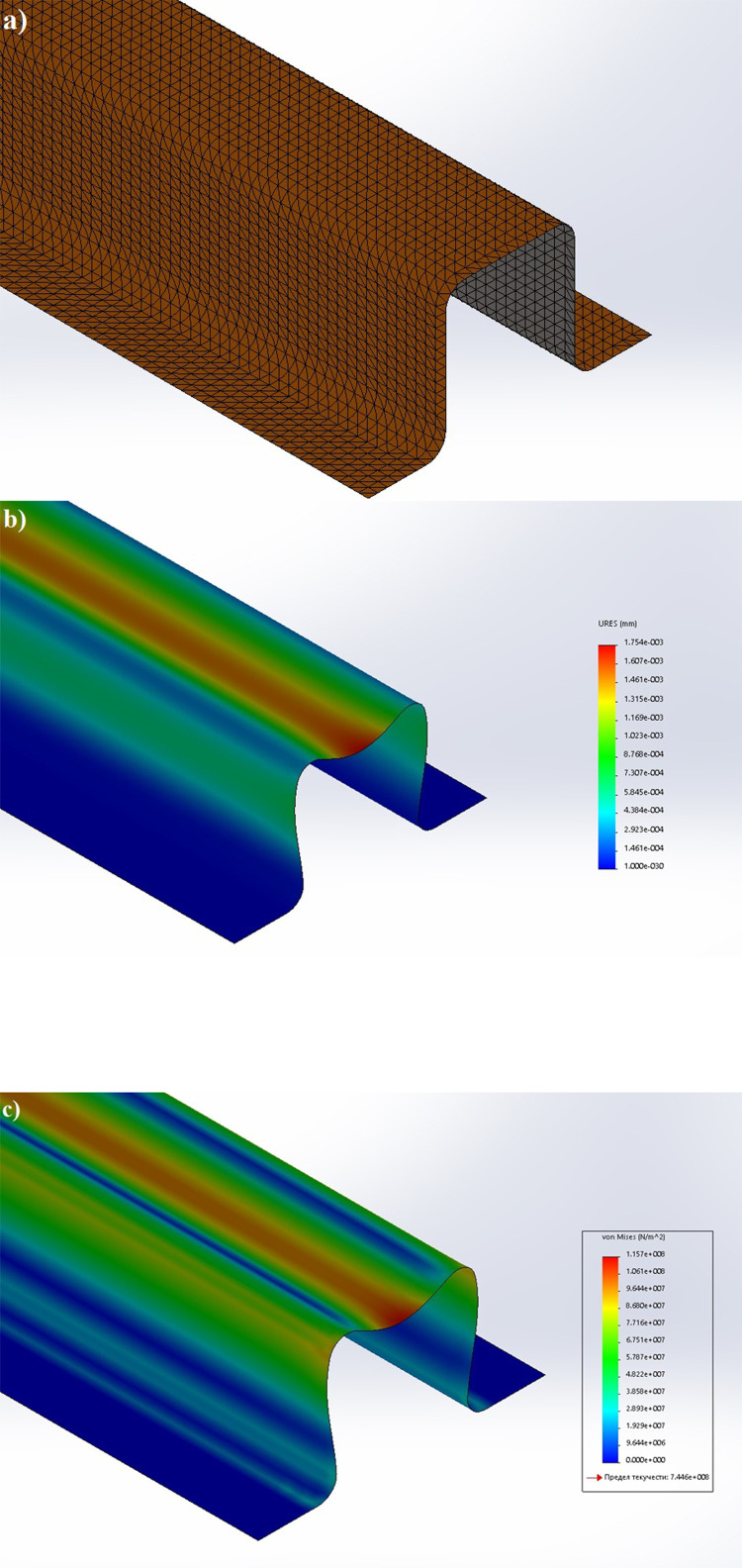
(a) Breaking a channel of the cathode part of bipolar plate into finite elements, (b) strain stress in the material of the single bipolar plate channel, (c) deformation of the single bipolar plate channel when a load is applied.

**Fig. 2:**
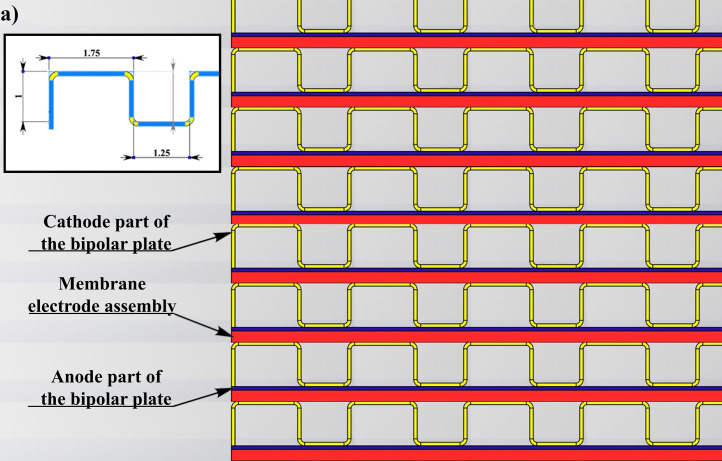
Fuel cell stack structure, the inset shows channels configuration of cathode part of the bipolar plate.

**Fig. 3:**
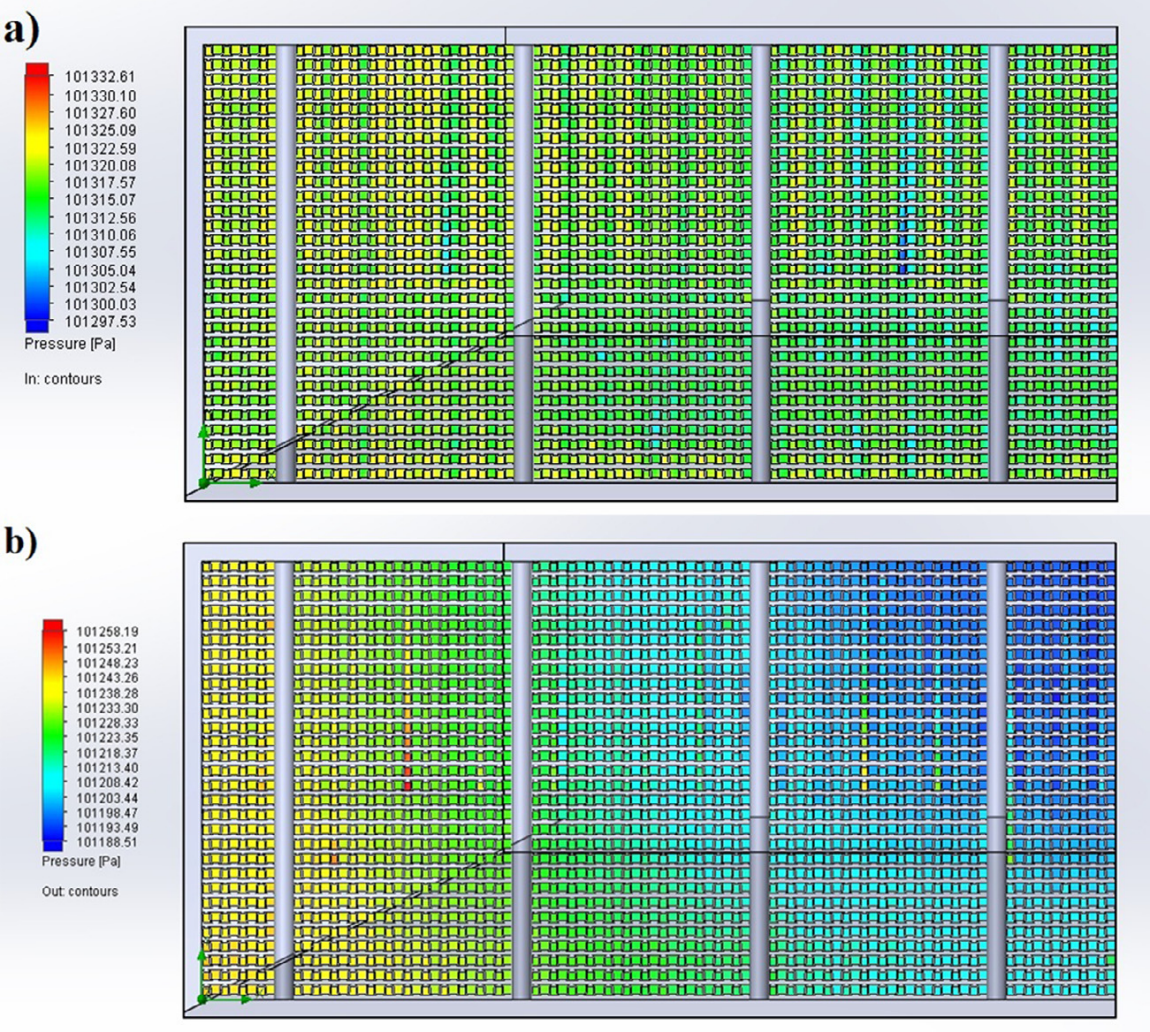
The oxidizing/cooling agent pressure field a) is at the inlet and b) is at the exit from the FC stack in cross section.

**Fig. 4:**
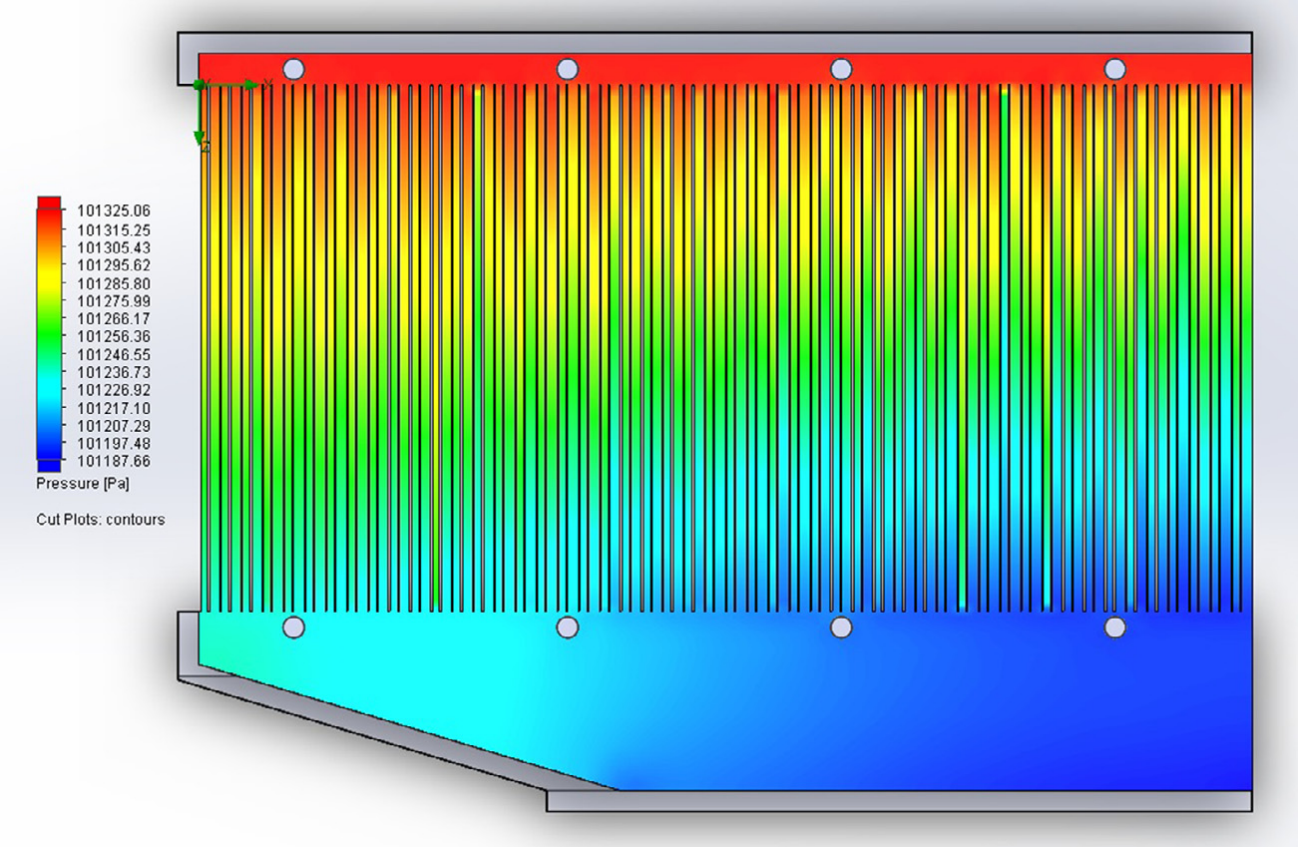
The oxidizing/cooling agent pressure field in the longitudinal section of the FC stack.

**Fig. 5:**
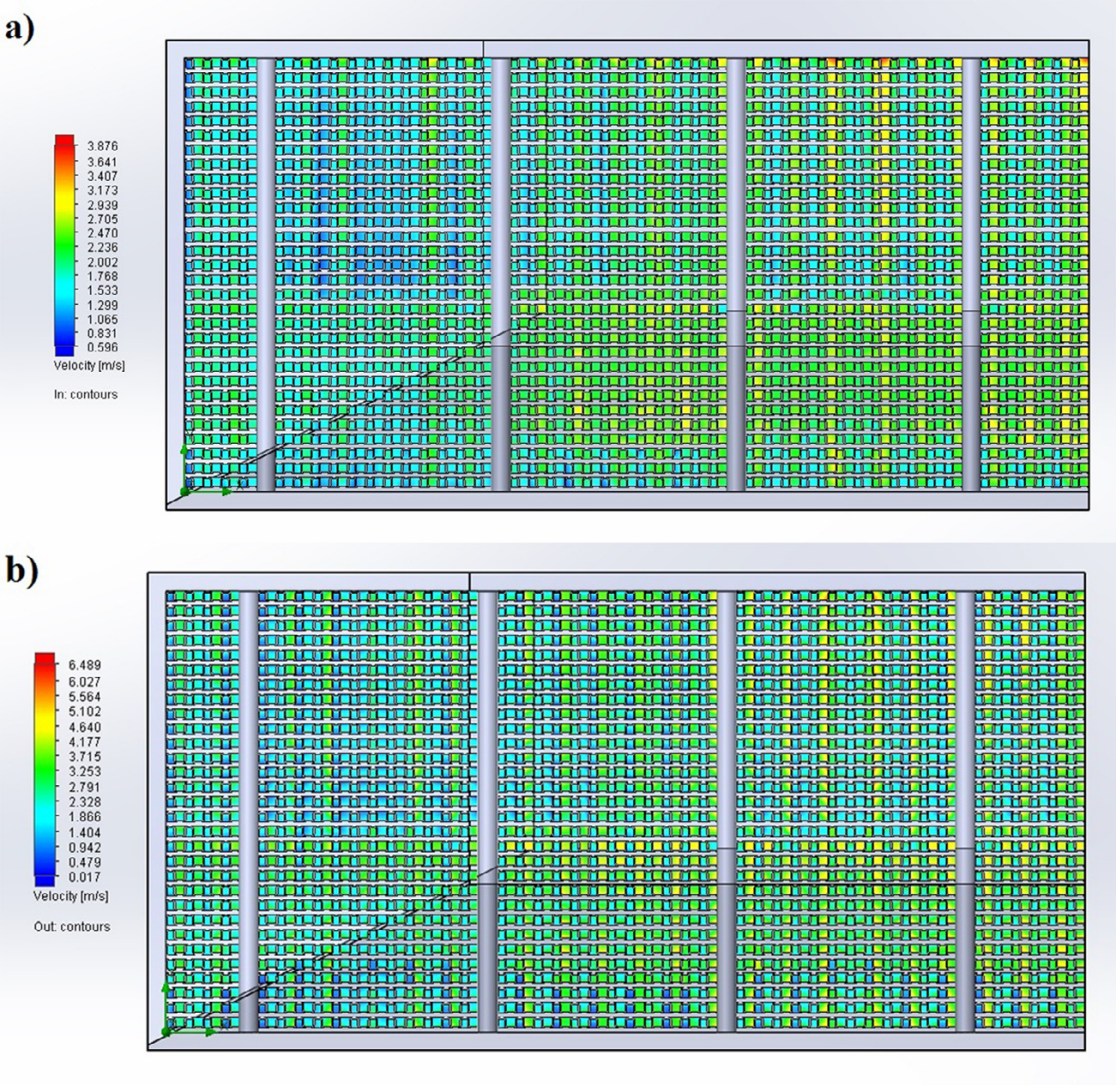
The velocity field of the oxidizing/cooling agent in the cross section of the FC stack a) is at the inlet, b) is at the outlet.

**Fig. 6:**
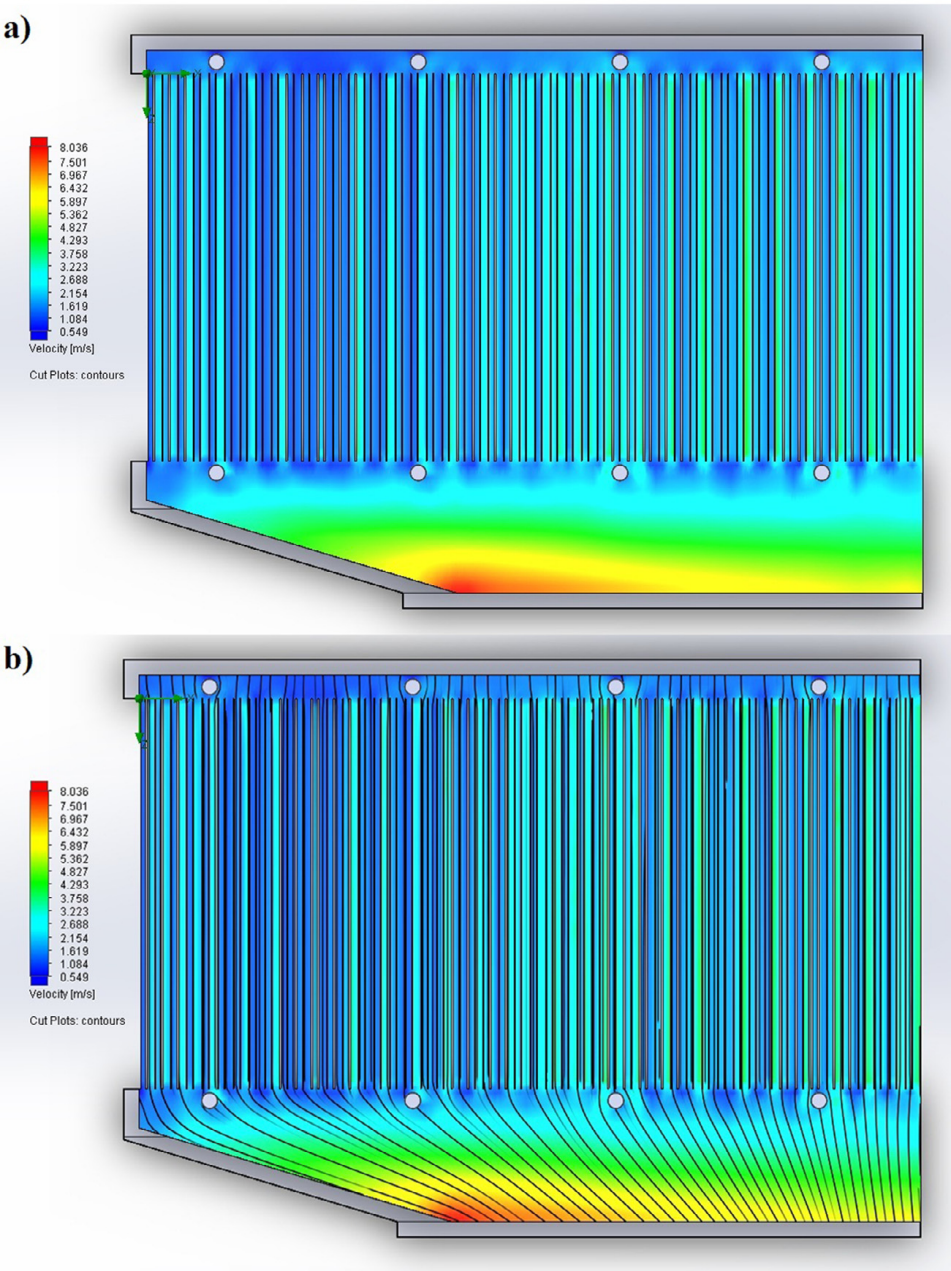
The velocity field of the oxidizing/cooling agent in the longitudinal section of the FC stack a) without streamlines and b) with streamlines.

**Fig. 7:**
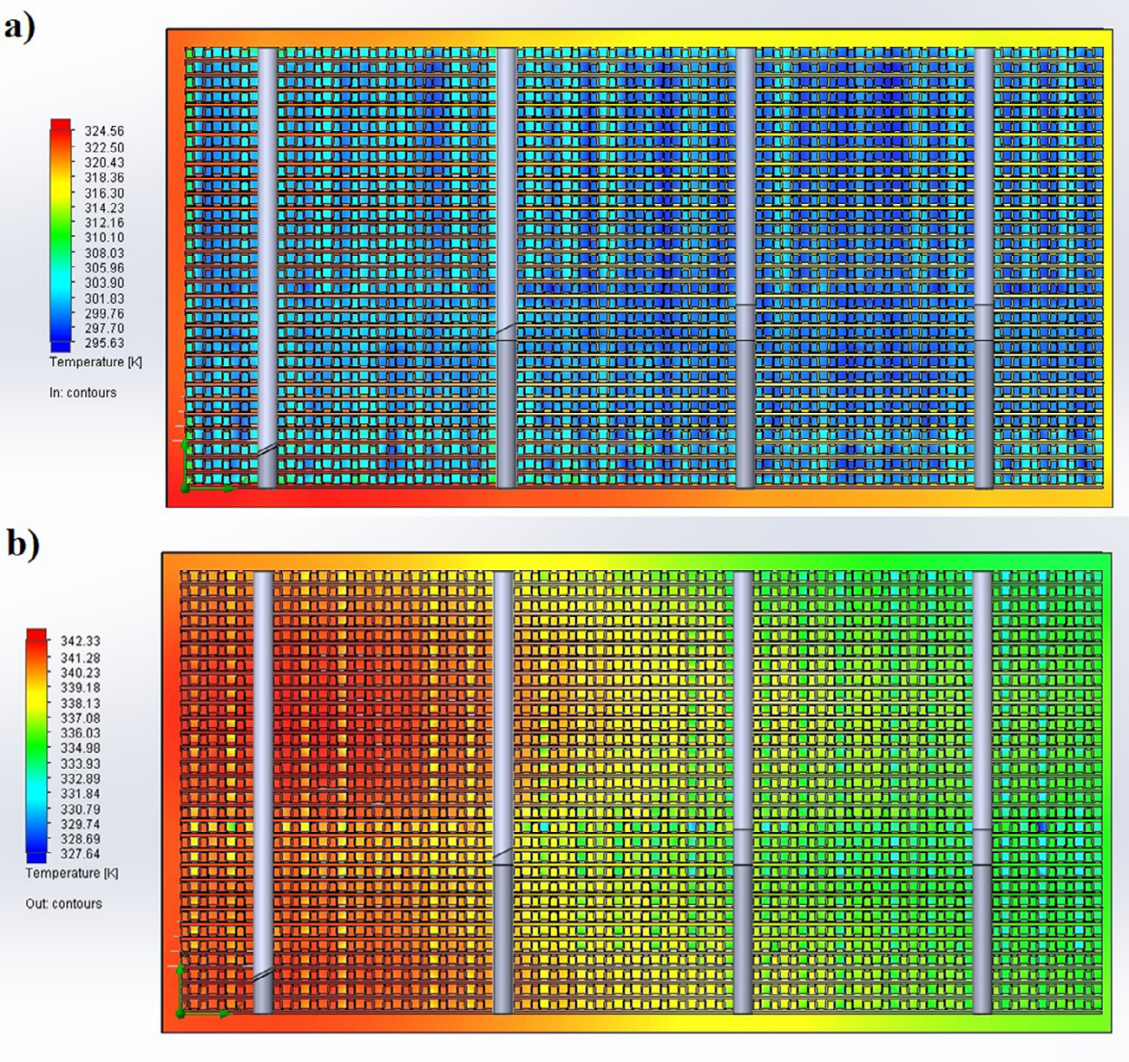
The temperature fields in the cross section a) at the inlet and b) at the exit from the FC stack.

**Fig. 8:**
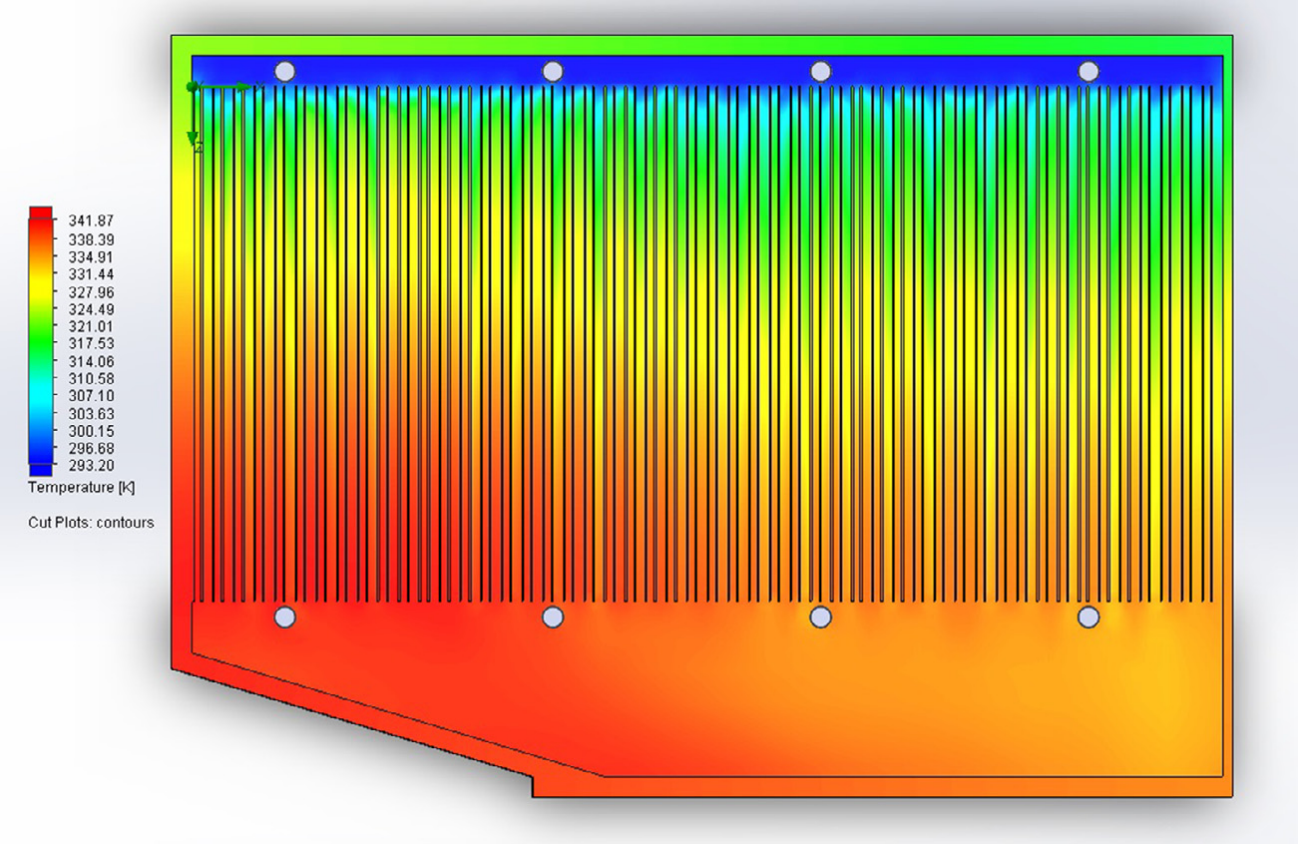
The temperature field in the longitudinal section of the FC stack.

**Fig. 9:**
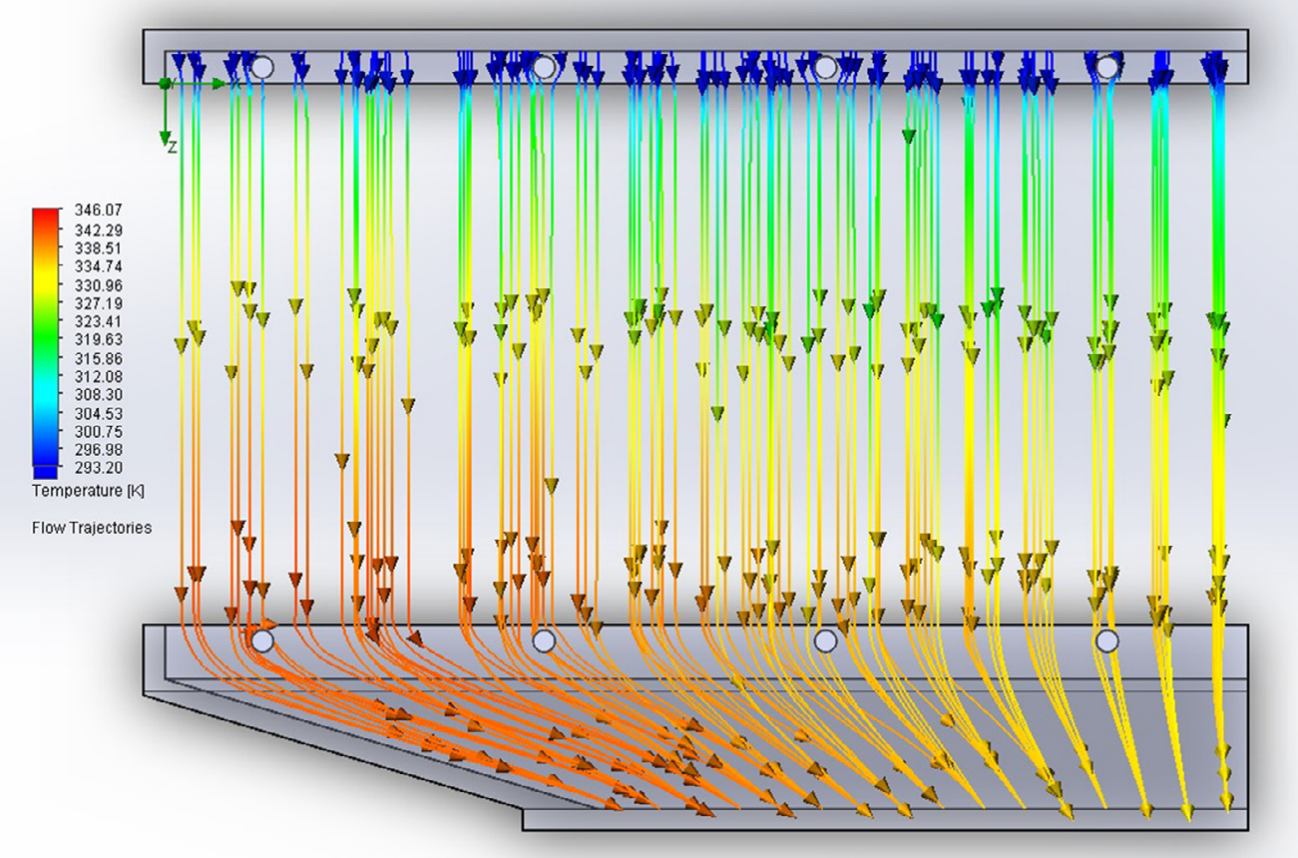
The trajectories of particles oxidizing/cooling agent flow with an indication of the temperature gradient in the FC stack section.

**Fig. 10:**
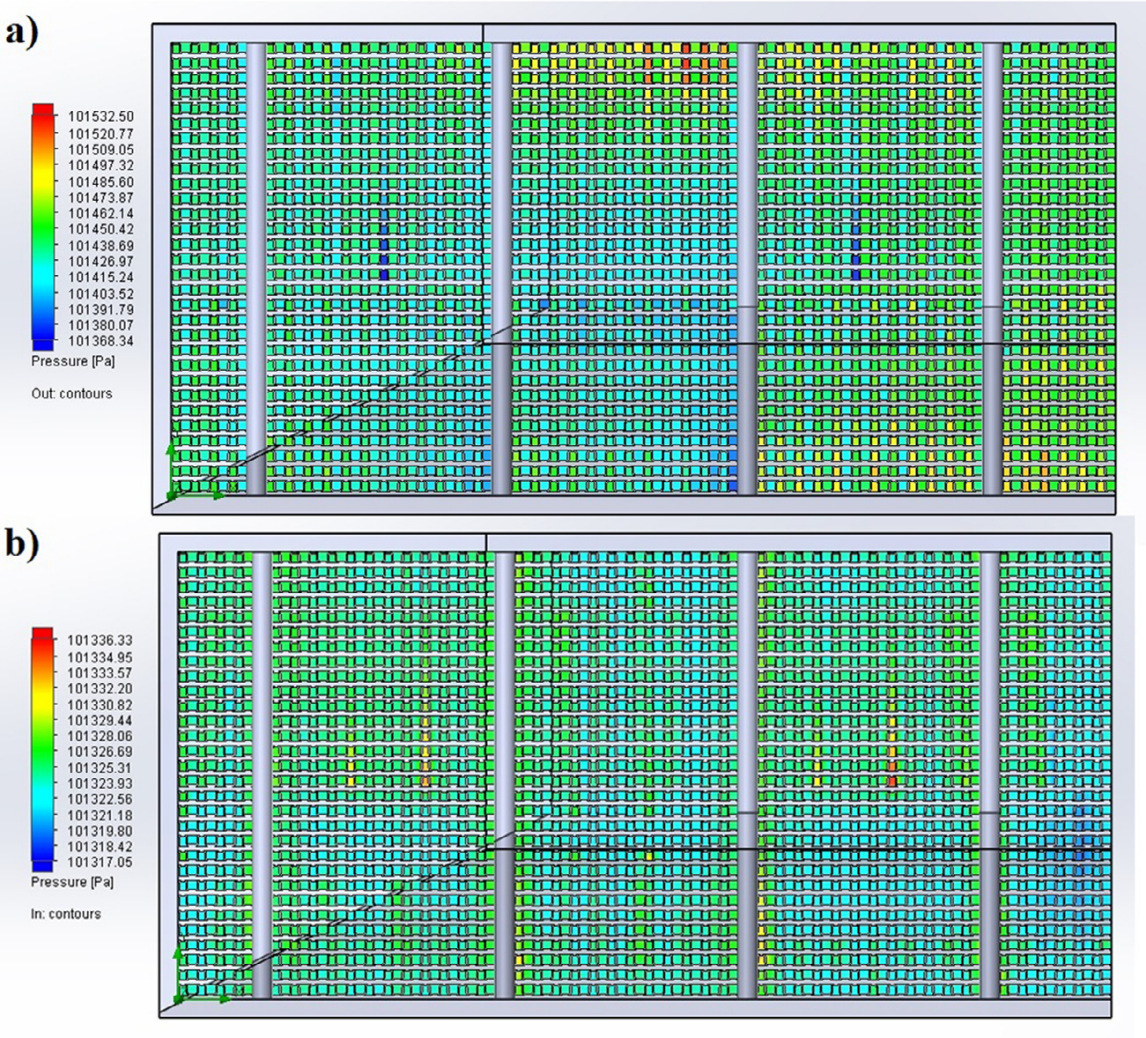
The oxidizing/cooling agent pressure field a) is at the inlet and b) is at the exit from the FC stack in cross section.

**Fig. 11:**
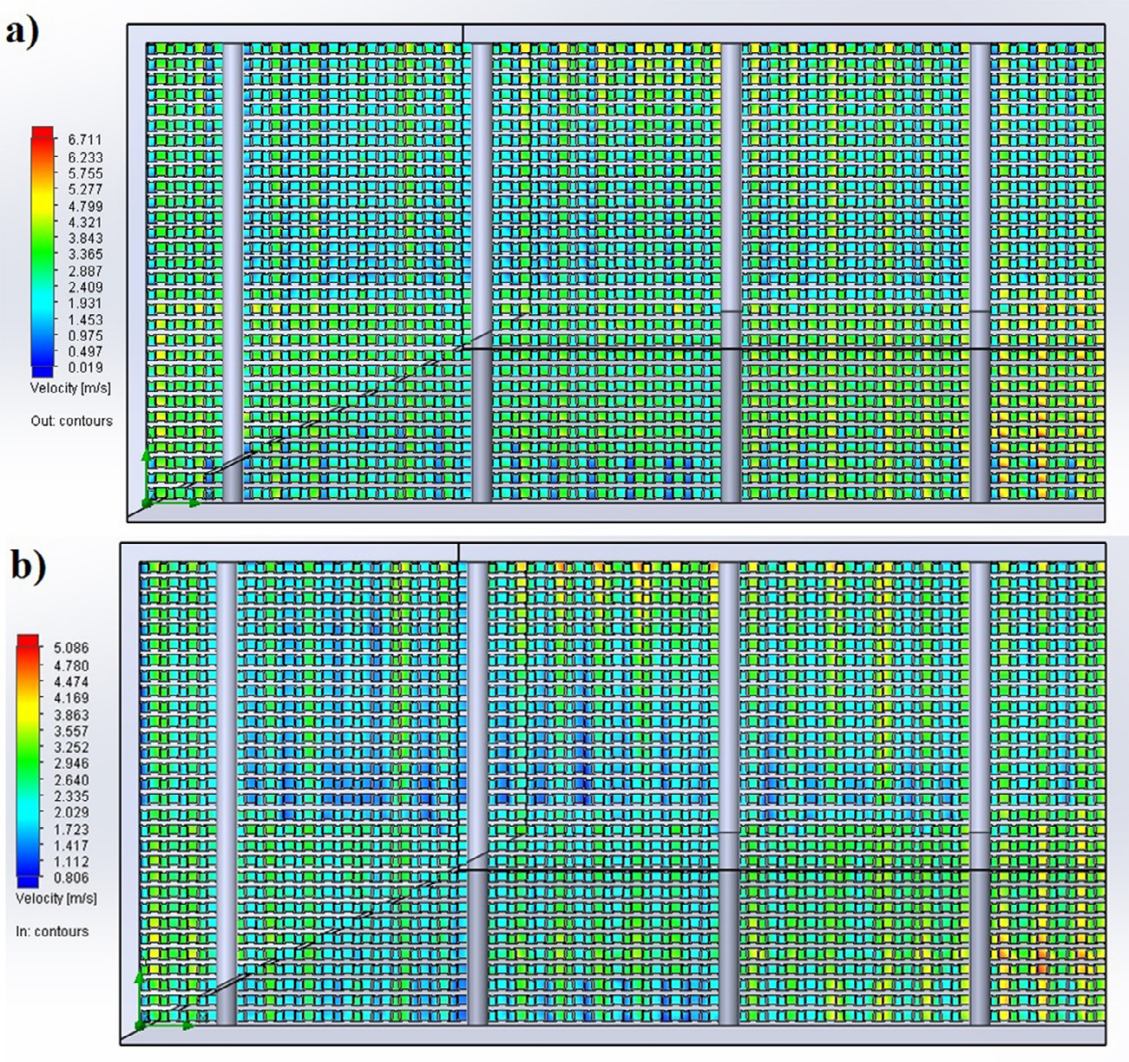
The velocity field of the oxidizing/cooling agent in the cross section of the FC stack a) is at the inlet, b) is at the outlet.

**Fig. 12:**
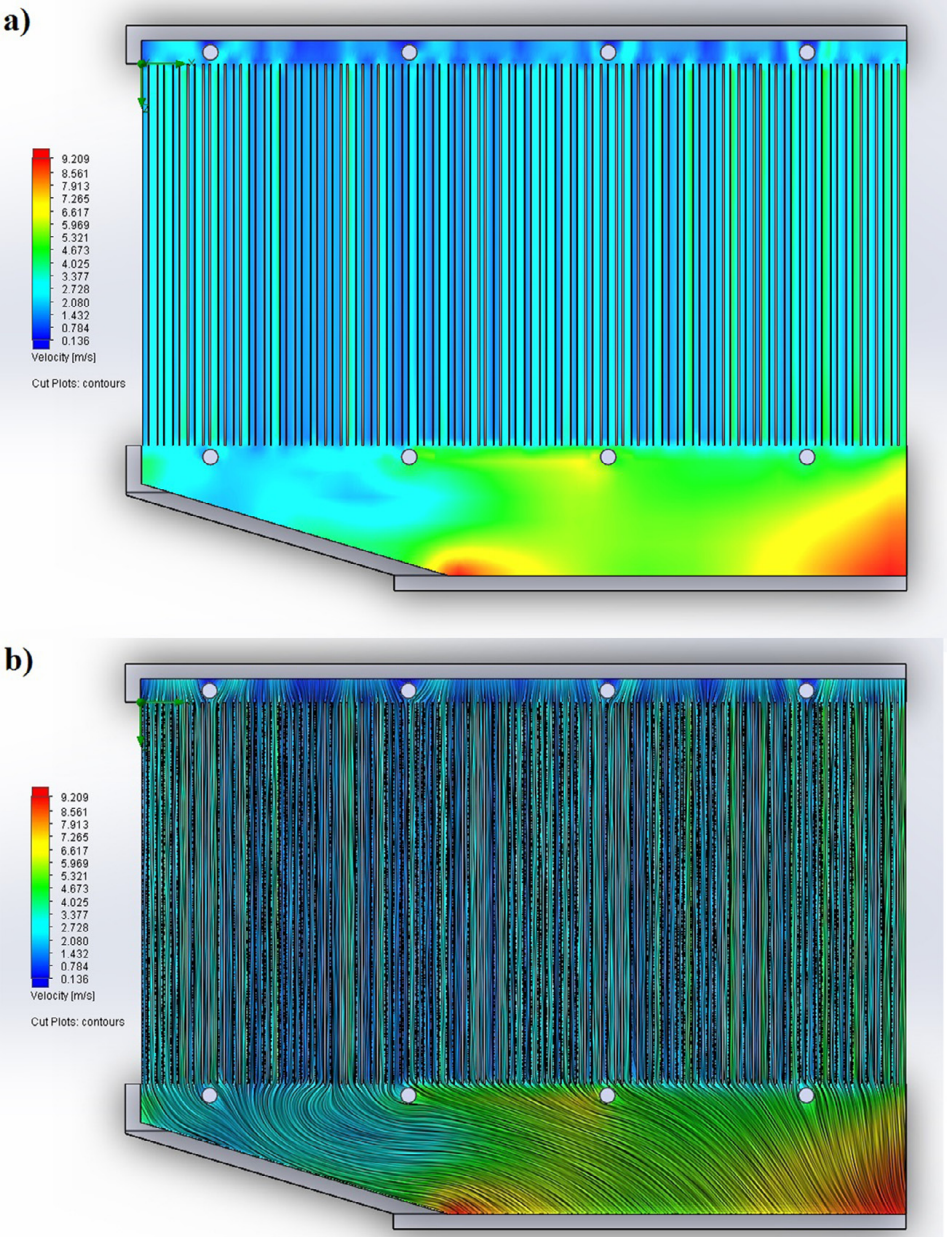
The velocity field of the oxidizing/cooling agent in the longitudinal section of the FC stack a) without streamlines and b) with streamlines.

**Fig. 13:**
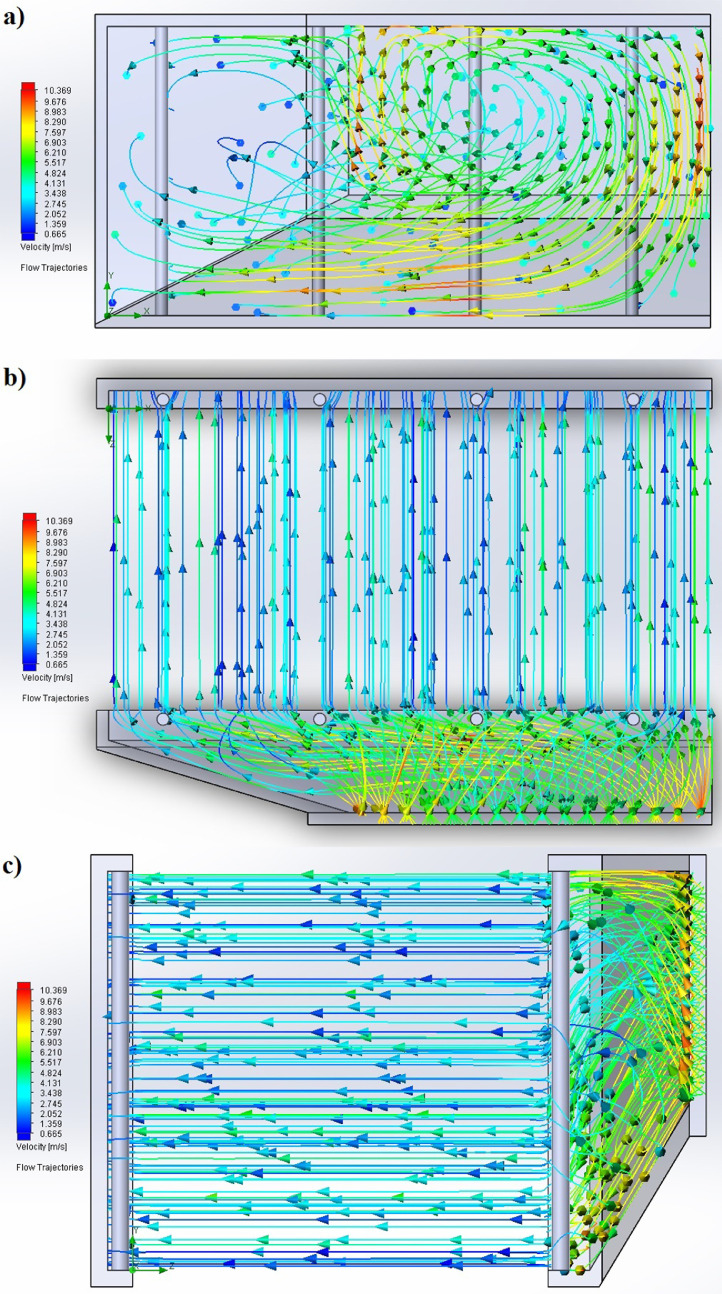
The trajectories of particles oxidizing/cooling agent flow in various sections: a) transverse, b) longitudinal and c) transverse (left view).

**Fig. 14:**
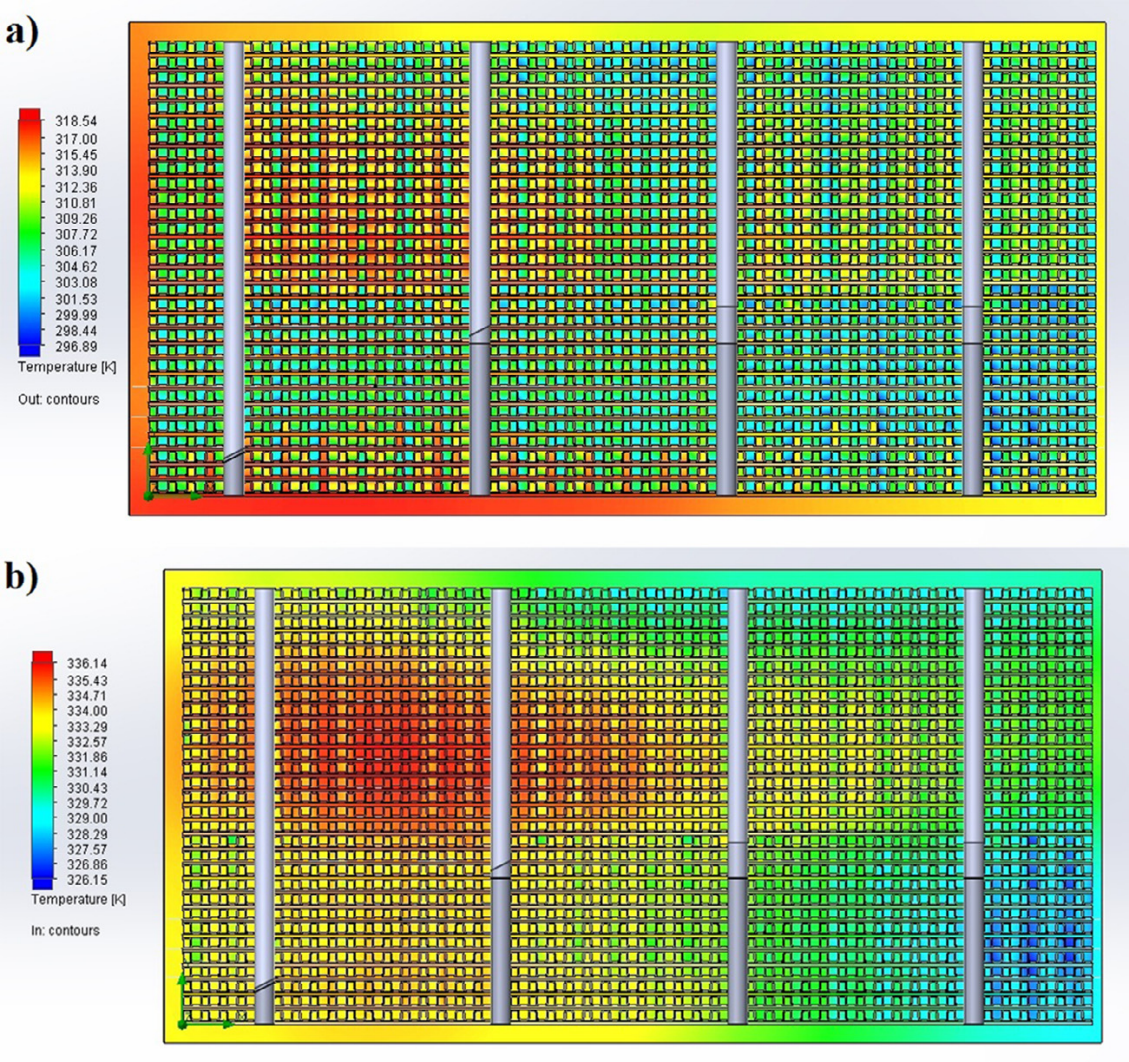
The temperature fields in the cross section a) at the inlet and b) at the exit from the FC stack.

**Fig. 15:**
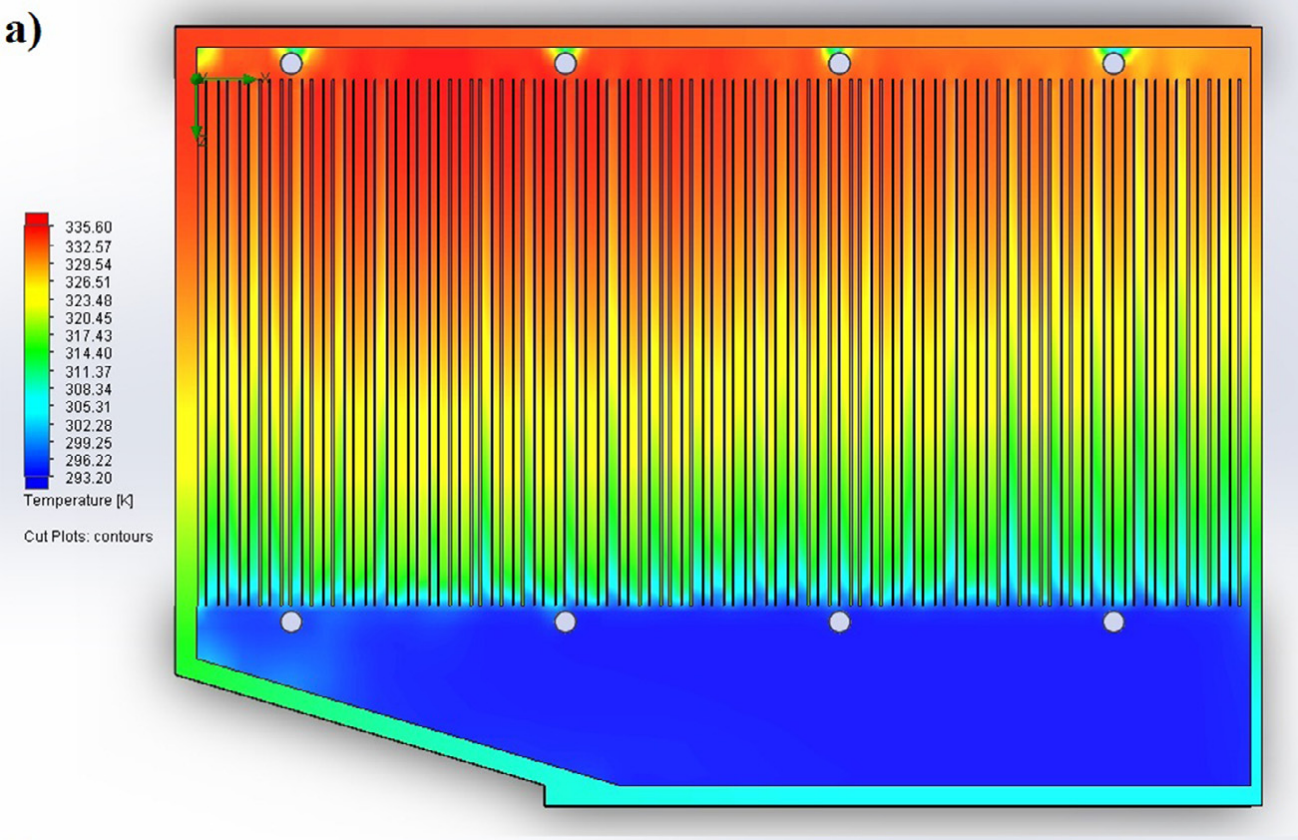
The temperature field in the longitudinal section of the FC stack.

The partition of the cathode side of bipolar plate (BP) channel into finite elements (a) and its deformation (b and c) are presented in Fig. 1. The strength calculation results of the cathode side of BP are presented in table 1. Fuel cell stack structure is presented in Fig. 2. The simulation was carried out for two variants of the oxidizer/cooling supply system location - at the outlet of the fuel cell stack (data presented in figures 3 - 9) and at the entrance to the fuel cell stack (data are shown in figures 10 – 15). Fig. 3 and Fig. 10 presents oxidizing/cooling agent pressure field at the inlet (a) and at the exit (b) from the FC stack. The oxidizing/cooling agent pressure field in the longitudinal section of the FC stack are presented in Fig. 4. The velocity field of the oxidizing/cooling agent in the cross section of the FС stack at the inlet (a) and at the outlet (b) of the FC stack are presented in Fig. 5 and Fig. 11. The velocity field of the oxidizing/cooling agent in the longitudinal section of the FC stack are shown in Fig. 6a and Fig. 12a. Moreover, Fig. 6b and Fig. 12b demonstrates the oxidizing/cooling agent rate field in the longitudinal section of the FC stack with streamlines. The temperature fields in the cross section at the inlet (a) and at the exit (b) of the FC stack are presented in Fig. 7 and Fig. 14. Fig. 8 and Fig. 15 are presented the temperature field in the longitudinal section of the FC stack. Fig. 9 demonstrates the trajectories of particles oxidizing/cooling agent flow with an indication of the temperature gradient in the FC stack section. The trajectory of the flow of particles in air flow in various sections shows in Fig. 13.

**Table 1:** Bipolar plate strength calculation results.

Yield strength	7.44634e+008 N/m^2^
Tensile strength	8.61e+008 N/m^2^
Compressive strength	8.3e+008 N/m^2^
Elastic modulus	1.103e+011 N/m^2^
Poisson's ratio	0.31
Mass density	4480 kg/m^3^
Shear modulus	4.8e+010 N/m^2^
Coefficient of thermal expansion	9e-006 /Kelvin
Strain (node 3348)	4,111*10^8^ N/m^2^
	2,881*10^−3^

## 2 Experimental Design, Materials, and Methods

### 2.1 Analysis of the strength and stability of the bipolar plates design

The strength of the bipolar plate depends on the strength of its cathode part, which has channels. The calculation was carried out for the cathode part of the bipolar plate. Strength calculation of a cathode part of bipolar plate was performed by the finite element method in the SolidWorks Simulation software. When assembling a fuel cell, a force will be applied to each plate during compression of the end plates [1]. The force acts perpendicular to the plane of the ridge of the channel. We accepted that the force is distributed evenly along the horizontal plane of the channels of the bipolar plate [2]. Figure 1 shows the partition of the single channel of the bipolar plate into finite elements (a) and channel deformation under loading (b and c). The single bipolar plate channel was divided into 33579 nodes and 16560 elements.

### 2.2 Thermo-fluid simulation of the PEMFC stack

Thermo-fluid simulations of the fuel cell stack were performed by the finite element method in the SolidWorks Flow Simulation program. The grid parameters, boundary conditions, heat-generating elements and their heat dissipation power were set for the simulation. The design of the fuel cell stack consists of the following elements:

- open-cathode fuel cells stack;
- number of fuel cells in stack - 30 units;
- fuel cell consists of a membrane electrode assembly and bipolar plate;
- titanium bipolar plate (size 40 • 237 mm) consist of two parts;
- cathode part of the bipolar plate has a channels configuration - width 1.75; height 1 mm; pitch - 1.25 mm;
- anode part of the bipolar plate has smooth configuration;
- two end-plates provided at the longitudinal, opposing ends of the fuel cells stack;
- plurality of tie rods, passing through a peripheral region of each end plate for positioning the fuel cell stack between the two end plates;
- two fans with confuser for supplying an oxidizing agent and cooling a fuel cell stack;
- fittings for supplying fuel (hydrogen), electrical leads.

The simulations have carried out for two variants of the oxidizer/cooling supply system location - at the outlet of the fuel cell stack (the system worked on air intake) and at the entrance to the fuel cell stack (the system worked on air injection). The simulations were carried out for a quarter of PEMFC stack, divided along the 2 axis of symmetry. In this case, a quarter of the stack was divided into 2114956 cells:

- cells in the fluid – 1047107 units;
- cells in the solid – 1067849 units;
- cells at the solid / fluid interface – 513138 units;

The simulations were carried out under the following conditions:

- oxidizer / cooling agent – air;
- oxidizer / cooling agent flow rate - 0.007 m^3^/s;
- heat emission as a result of fuel oxidation - 50% of the nominal stack power.
- heat transfer coefficient from FC stack to outside – 0;
- atmospheric pressure - 101.325 Pa;
- ambient temperature - 20°C.

The oxidizer / cooling supply system are two fans. It may causes disturbances and swirls of the air flow due to rotatation of the blades that not always have an optimal profile. In addition, the tie rods of PEMFC stack are opposite the entry and exit of the set of FC stack, which may contribute to disturbance and swirl of the air flow. The product of the reaction of the fuel cell is water. Self-humidification of the fuel cell occurs during operation in a steady-state process. The thermophysical properties of air at a calculated temperature range (20°C – 50°C) are practically independent of humidity [3]. The simulation was carried out for the most severe operating PEMFC conditions of zero air humidity. The fuel cell technology program of U.S. Department of Energy claims that, commencing 2011, PEMFSs achieved 50% efficiency [4]. Based on the above, some assumptions were used in the simulations. The following list details the major assumptions used in developing this model:

- Stationary steady-state process FC operating;
- Single phase air flow;
- Air is uniform in the stack;
- Air temperature inside the FC stack does not affect the thermophysical properties of the air;
- The effect of air humidity on electrochemical processes is not taken into account;
- Dry (zero humidity) air;
- Heat emission as a result of fuel oxidation is equal to 50% of the nominal stack power;

#### 2.2.1 The oxidizer/cooling supply system is located at the outlet of the fuel cell stack

 

### 2.3 The oxidizer/cooling supply system is located at the entrance to the fuel cell stack

Similar calculations were made with a fan located at the entrance to the FC stack.

## Declaration of Competing Interest

The authors declare that they have no known competing financial interests or personal relationships which have, or could be perceived to have, influenced the work reported in this article.

## Appendix Supplementary materials

Image, application 1
